# Bringing Light Into the Dark: Associations of Fire Interest and Fire Setting With the Dark Tetrad

**DOI:** 10.3389/fpsyg.2022.876575

**Published:** 2022-06-27

**Authors:** Caroline Wehner, Matthias Ziegler, Simon Kirchhof, Lena Lämmle

**Affiliations:** ^1^Psychological Assessment, Humboldt-Universität zu Berlin, Berlin, Germany; ^2^Health Psychology, Medical School Hamburg, Hamburg, Germany

**Keywords:** Dark Triad, Dark Tetrad, sensation seeking, fire interest, fire setting

## Abstract

Fire setting is a significant problem for society, costing many human lives and causing great property damage. One important risk factor of fire setting observed in forensic samples is fire interest. However, less is known about the relationship of fire interest and fire setting to other variables such as personality traits in subclinical samples. In this study, we observed the relationship of potentially important personality traits with fire interest and fire setting in a sample of *N* = 222 students. In addition to zero-order correlations, we calculated path models and a logistic regression including all predictor variables. From the Dark Tetrad, consisting of psychopathy, narcissism, Machiavellianism, and three facets of sadism, psychopathy, and physical sadism were found to be associated with fire interest and fire setting. Furthermore, vicarious sadism was associated with fire interest. The other Dark Tetrad traits and four sensation seeking facets did not substantially add to the predictions. This confirms the results of previous studies with clinical and forensic samples with psychopathy and sadism as relevant predictors for fire interest and fire setting. Our results also provide evidence for viewing sadism as the multidimensional construct discriminating between vicarious and other forms of sadism, for the distinction of psychopathy and Machiavellianism, and for the Dark Tetrad being linked to object related violence.

## Introduction

Fire has always played an important role in humanity, either as a source of warmth and light, a means for cooking in traditional societies, or as a marker for social events and a source for entertainment in Western countries (Fessler, 2006). However, tragic cases like the recent wildfires in California and Greece or the Notre-Dame fire in 2019 also bring the destructive potential of fire to the public consciousness. When used deliberately or by accident fire causes severe damages to both humans and property. For example, in 2018, there were about 1,318,500 fires in the USA of which 25,000 were intentionally set. The fires caused 3,655 deaths and destroyed property worth US$25.6 billion. A total of 350 deaths and a damage of US$593 million were caused by proven intentionally set fires (Evarts, 2019). Due to this destructive potential, it is necessary to explore fire setting behavior and investigate which factors lead an individual to it. Research has identified several psychological vulnerabilities that are qualified as potential risk factors. One of these is fire interest, the fascination with fire and fire setting (Dickens et al., 2009; Gannon and Barrowcliffe, 2012). High amounts of fire interest and especially interest in severe fires could be observed in convicted fire setters (Gannon et al., 2013; Tyler et al., 2015) and the latter also differentiated between fire setters and other offenders (Gannon et al., 2013). Overall, previous research on fire setting research has mainly investigated fire setting behavior and fire interest in forensic or clinical populations (e.g., Ducat et al., 2013; Thomson et al., 2017; Allely, 2019; Wyatt et al., 2019), lacking to address fire interest in non-forensic and non-clinical populations. At first glance, this might make sense, given that the life-time prevalence for fire setting in the general population ranges only from 1 to 1.3% (Blanco et al., 2010). Nevertheless, this would still make about 3.3 million fire setters in the USA alone. Moreover, many studies and theoretical developments in recent years have stressed the importance of integrating findings from subclinical personality and pathological personality characteristics (Hopwood et al., 2015). A better understanding about the relationship between dark traits and fire setting can potentially inform prevention efforts or theories on how the pathology develops. Therefore, this study explores the relationship between selected personality traits and fire interests adding to previous research on the latter mainly stemming from clinical and forensic psychology.

There are several individual difference variables which are likely to explain variance in fire interest and fire setting from a theoretical point of view and based on previous empirical findings. Two traits which have been hypothesized to be associated with fire interest and fire setting are impulsivity and thrill-seeking (Gannon et al., 2012). The link between fire setting and impulsivity has also been shown empirically (Hoerold and Tranah, 2014; Wyatt et al., 2019). Since psychopathy includes impulsivity as one of its central aspects (e.g., Paulhus and Williams, 2002; Jones and Paulhus, 2011; Verschuere and te Kaat, 2019), we considered it a trait potentially relevant for the prediction of fire interest and fire setting. Other relevant individual difference variables that have not been investigated in the context of fire interest and setting include those of the Dark Tetrad traits and sensation seeking.

Consequently, our goal was to investigate the nomological net of fire interest and fire setting in a subclinical sample. As the Dark Tetrad variables overlap (Sleep et al., 2017; Blötner et al., 2021), bivariate correlations with criteria can be misleading. Multivariate approaches are used to identify specific relations between the Dark Tetrad components and criteria. At the same time, such analyses lead to the risk of suppression effects. Therefore, the combination of viewing bivariate correlations and regression weights yields the best foundation for a sound interpretation of the relations. Hence, we tested if the Dark Tetrad (psychopathy, narcissism, Machiavellianism, and sadism) and sensation seeking are related to fire interest and fire setting using correlations as well as path models to identify exclusive contributions. In the following, the literature surrounding fire interest and fire setting and their possible links to the Dark Tetrad as well as sensation seeking are further examined.

### Fire Interest and Fire Setting

Fire interest describes a general interest in and fascination with fire and fire setting, while fire setting is understood as the intentional act of setting a fire. An extreme form of fire interest and fire setting is pyromania, which is classified in DSM-5 as an impulse control disorder. Pyromania is characterized by high fire interest but also multiple occasions of fire setting with an experience of tension and arousal before, and relief after the act (American Psychiatric Association, 2013; Ó Ciardha et al., 2017). Due to the relatively narrow diagnostic criteria, pyromania is rarely diagnosed (Gannon et al., 2012; Ó Ciardha et al., 2017) and while individuals with a pyromania diagnosis will always show high fire interest and fire setting behaviors, most individuals with high fire interest and most fire setters are unlikely to be diagnosed.

A theory which incorporated fire interest as an important factor for fire setting is the Multi-Trajectory Theory of Firesetting (M-TTAF; Gannon et al., 2012; Tyler and Gannon, 2020; see Dalhuisen et al., 2017 for an empirical test). The M-TTAF describes how psychological vulnerabilities and other factors such as cultural or developmental aspects as well as situational context and social learning can lead to fire setting. The authors suggested four trajectories (antisocial, grievance, fire interest, and need for recognition) of fire setting which may develop due to differing risk factors and contexts (e.g., life events). A fifth trajectory (multifaceted) entails combinations of the other four trajectories. The fire interest trajectory is defined by a high fire fascination, impulsivity, and fire supporting attitudes (e.g., “I can control fire”). Thrill, stress, and boredom are named as potential motivators of this trajectory (see also Grant and Kim, 2007; Butler and Gannon, 2015) and fire is assumed to be a means of coping with these feelings (Gannon et al., 2012). Alternatively, the authors state that fire setting itself could be experienced as pleasurable due to the intense affective or sensory stimulation. Gannon et al. (2012) suggested that fire interest develops from an interaction of social learning, cultural influences, and classical conditioning. In conclusion, it is not theorized that fire setting is always motivated by fire interest but fire interest can play a vital role in some fire setters and might also be present in individuals in the multifaceted trajectory.

In the following, we discuss the potential relationships of the Dark Tetrad traits and sensation seeking with fire interest and how these might fit to the trajectories of the M-TAFF.

### The Dark Tetrad and Fire Interest

As outlined above, there are several studies which have suggested that the Dark Tetrad traits may be suitable in predicting fire interest and fire setting. In the following, we will describe the empirical evidence for each trait. It is important to note that the literature on fire setting is overall rather scarce (see also Gannon et al., 2012) and most studies were conducted in a forensic or clinical setting with unclear generalizability to non-clinical populations.

#### Psychopathy

There exists conflicting evidence regarding the prevalence of psychopathy in the population of fire setters. Some researchers suggested that psychopathy is an uncommon diagnosis, with a prevalence of 1.5% (*n* = 3; Dresdner Cid and Folino, 2017) and others have found that scores are on average not higher in offenders who have set fires compared to offenders who have not set fires (Labree et al., 2010). However, Thomson et al. (2015) showed that although psychopathy was not related to recidivistic arson offenses, an amount of 16.3% (*n* = 21) of arson offenders had scores ≥ 25 on the Psychopathy Checklist Revised. Although evidence regarding the prevalence of clinical psychopathy in the fire setting population is mixed there is evidence that several facets captured by subclinical psychopathy measures are present in fire setters. Jones and Paulhus (2014), for example, identified impulsivity and callousness as central attributes operationalized in their Short Dark Triad measure (SD3). High levels of impulsivity have often been observed in fire setters (Räsänen et al., 1996; Gannon and Pina, 2010; Labree et al., 2010; Hoerold and Tranah, 2014) and are also part of the fire interest trajectory of the M-TAFF (see above). The evidence for callousness is less pronounced. A study with adolescents showed higher callousness in fire setters compared to other antisocial adolescents and a control-group (Hoerold and Tranah, 2014). In a further adolescent sample, callous-unemotional behavior was only related to fire setting in individuals with low antisocial behavior (Watt et al., 2015). In the Self-Report Psychopathy Scale-III (SRP-III; Paulhus et al., 2009) interpersonal manipulation was identified as a further central facet of psychopathy. To the best of our knowledge, there is no research which has observed the relation of interpersonal manipulation and fire setting. Given the broad evidence from clinical research, it seems worth to include psychopathy into the analyses of potential correlates of fire interest and fire setting in subclinical samples. In particular multivariate analyses, controlling of other dark traits, as was done in this study, can help to gain information on the overlap in variance of psychopathy and fire interest and fire setting.

#### Machiavellianism

Immorality, manipulation, a duplicitous interpersonal style, and a focus on personal gain have been described as central for Machiavellianism (Christie and Geis, 1970; Muris et al., 2017). Several antisocial behaviors, which have been identified to be common in fire setters (Blanco et al., 2010), fit to the aforementioned characteristics of Machiavellianism such as frequent lying, using a false name, scamming others for financial gain, and blackmailing. Fraud, which could be added to these behaviors, has also been identified as a common motive for arson (Geller, 2008). Using fire setting for financial gain also fits to the antisocial trajectory proposed in the M-TTAF (Gannon et al., 2012). Within the multifaceted trajectory of the M-TTAF, fire setters' fraudulent behavior could potentially also go along with fire interest. Even though the evidence for the relation of fire setting and fire interest to Machiavellianism is not as strong as evidence supporting the relation with psychopathy, the identified behavioral correlates make Machiavellianism an interesting variable to observe in relation to fire. A further reason for adding Machiavellianism is the often found overlap in the nomological networks of psychopathy and Machiavellianism (e.g., Vize et al., 2018). It would be interesting to see whether the overlap also holds in the prediction of fire interest and fire setting. For example, for vandalism, the correlative association tended to be higher for psychopathy, *r* = .54, than for Machiavellianism, *r* = .31, and in a regression including the Dark Tetrad, psychopathy did (β = .28) and Machiavellianism did not predict (β = −.02) vandalism (Pfattheicher et al., 2019) indicating a partial overlap of the traits but with psychopathy explaining variance not shared with Machiavellianism. Thus, this study by looking at Machiavellianism in relation with fire interest and fire setting will not only help to explore the nomological network of these behaviors but also help to further elucidate the overlap between Machiavellianism and psychopathy.

#### Narcissism

Key characteristics of subclinical narcissism^1^ are grandiosity, entitlement, and an egotistic admiration of the own attributes (Jones and Paulhus, 2014; Muris et al., 2017). With regards to fire setting, Campbell (2009) hypothesized that there exists a type of fire setter for whom narcissism is a defining trait with the characteristics, such as grandiosity, entitlement, and lack of self-control. In a sample of forensic patients identified as arsonists, who were diagnosed with a not otherwise specified personality disorder, narcissistic traits were most common (32%; Wilpert et al., 2017). However, there is also evidence suggesting that narcissism was not more common in fire setters than in a criminal control group (Ó Ciardha et al., 2015a). Narcissism is also not directly incorporated in the trajectories of the M-TTAF (Gannon et al., 2012), although those authors hypothesized that narcissism may play a role in the need for recognition trajectory by facilitating the fire setters' need for social recognition and status enhancement. Thus, while the relation with narcissism might not be unique to fire setting, there is a reason to assume that such a relation does exist.

#### Sadism

Sadism, in its subclinical form, is understood as the enjoyment of causing or observing the suffering of others (Paulhus et al., 2020). It has been conceptualized with the three facets, namely, direct verbal, direct physical, and vicarious sadism (Buckels and Paulhus, 2013). The first two facets have also been combined to direct sadism (Paulhus and Jones, 2015). While direct sadism is focused on actively harming others (e.g., by physical force or by using insults), vicarious sadism describes the enjoyment of violence committed by others (e.g., in violent media or sports). Recent evidence showed a connection of sadism and vandalistic acts carried out for pleasure, with direct sadism as the most influential predictor (see also Burton et al., 2012; Pfattheicher et al., 2019). This could also explain a link between direct sadism to fire interest and fire setting. Other findings suggest that fire interest might possess, like vicarious sadism, a similar vicarious need to perceive destruction: Fire setters with high fire interest were found to be watching their own fire if possible (Tyler and Gannon, 2017) and patients with a pyromania diagnosis were interested in driving toward fires in their vicinity to watch them (Grant and Kim, 2007). Within an offender sample, female fire setters and other female offenders showed similar levels of sadism (about 12% had a sadistic disorder and about 9% had increased sadism levels) but male fire setters tended to be more sadistic (18% had a sadistic disorder and another 18% had increased sadism levels; Alleyne et al., 2016). Similar to psychopathy, sadism was found to be related to impulsivity (March et al., 2017; Blötner et al., 2021) which also may explain a link to fire interest. Overall, the previous evidence suggests a link between sadism and fire interest and fire setting, which may differ in strength depending on the sadism facet.

### Sensation Seeking and Fire Interest

Sensation seeking can be described as the preference for novel, intense, and varied experiences and the willingness to take risks for the sake of these experiences (Zuckerman, 1994). It has been found to be associated with criminal behavior, such as shoplifting or selling drugs, and damaging property through, for example, vandalism and arson (Zuckerman, 2007). In the M-TTAF (Gannon et al., 2012) sensation seeking is represented through thrill seeking as a potential motivator in the fire interest trajectory. Thus, thrill seeking as a facet of sensation seeking (Zuckerman et al., 1978) is a potential connection to fire interest. Another connection may represent the close relation of sensation seeking and impulsivity (Whiteside and Lynam, 2001). As mentioned earlier, the relationship of impulsivity and fire setting was also shown empirically (Hoerold and Tranah, 2014; Wyatt et al., 2019) and is part of the M-TTAF (Gannon et al., 2012). Doley et al. (2011) mentioned that boredom is related to fire setting in adult offenders, which is covered by a further sensation seeking facet (boredom susceptibility; Zuckerman et al., 1978). The authors argue that this is because many arsonists described boredom as an important motive for committing arson, as the fire setting may support the relief of boredom (Doley et al., 2011). Thus, sensation seeking at the facet level was added as potentially relevant associations to fire interest and fire setting.

In line with the aforementioned research, we hypothesized that the Dark Tetrad and sensation seeking are related to fire interest and fire setting.

## Methods

### Participants and Procedure

In this study, we recruited psychology and medical students from German universities, who filled an online questionnaire. The former were compensated with course credit and all participants could participate in a lottery. In total, we obtained a sample of 239 individuals, with 17 cases being excluded due to missing at least one whole questionnaire. This resulted in a final sample of *N* = 222 with 46% of the participants identifying as female. Participants' age ranged from 18 to 60 (*M* = 24.7; *SD* = 6.1). Seven percent (*n* = 16) reported that they had set a fire in the past with ages ranging from 23 to 30 (*M* = 25.7; *SD* = 2.1).

### Measurements

#### Fire Interest and Fire Setting

Fire interest was measured with the 14-item Fire Interest Rating Scale (FIRS; Murphy and Clare, 1996). Items describe everyday fires (e.g., “Watching an ordinary coal fire in an ordinary house.”) and also severe fires (e.g., “Seeing a hotel on fire on the TV news.”) which were rated by the participants on a scale from 1 (*absolutely horrible or most upsetting possible*) to 7 (*lovely, very exciting, or very nice*). In our data, the FIRS showed an acceptable internal consistency estimate of α = .78. The FIRS has been shown to successfully discriminate between fire setters and non-fire setters (Ó Ciardha et al., 2015b). We used one additional item (“Have you ever set a fire?”) to assess whether or not the participants ever had intentionally set fire (FS; fire setting). To ensure the correct understanding of the item, we added a fill-in note saying that the question does not apply to socially accepted lighting of combustibles to light fire places, bonfires, and Easter fires or the like.

#### Dark Triad

The Dark Triad was measured with the German version of the Short Dark Triad (SD3; Wehner et al., 2021) which consists of 28 items in which participants rated on a scale from 1 (*disagree strongly*) to 5 (*agree strongly*). The three subscales assessed Machiavellianism (“Make sure your plans benefit yourself, not others;” α = .73), narcissism (“People see me as a natural leader;” α = .70), and psychopathy (“Payback needs to be quick and nasty;” α = .76).

#### Sadism

Sadism was measured with the Comprehensive Assessment of Sadistic Tendency scale (CAST; Buckels and Paulhus, 2013). The CAST consists of 18 items, which were rated on a scale from 1 (*strongly disagree*) to 7 (*strongly agree*). The instrument measures direct verbal sadism (“I have purposely tricked someone and laughed when they looked foolish;” α = .82), direct physical sadism (“I enjoy physically hurting people;” α = .83), and vicarious sadism (“In video games, I like the realistic blood spurts;” α = .80). Internal consistencies are comparable to those found by Plouffe et al. (2018).

#### Sensation Seeking

Sensation seeking was measured with the Sensation Seeking Scale V (SSS-V; Zuckerman et al., 1978; Zuckerman, 1996) consisting of 40 items. The construct was measured in a binary forced choice format, where participants decided between two options A and B (“A: I like “wild” uninhibited parties;” “B: I prefer quiet parties with good conversation”). The SSS-V included four subscales, thrill and adventure seeking (α = .78), boredom susceptibility (α = .50), disinhibition (α = .68), and experience seeking (α = .48). Internal consistencies are comparable to those found by Beauducel et al. (2003).

All used instruments except for SD3 were translated to German by L.L. and S.K. based on a back-translation procedure and can be found in the codebook on the OSF (https://osf.io/z9rgd/).

### Statistical Analyses

All analyses^2^ were conducted in R version 3.6.3 (R Core Team, 2020). Data and code can be found on the OSF. We first estimated the measurement model for each (sub)scale using WLSMV estimators. In addition, we estimated a common (bifactor) model including all SSS-V subscales, as requested by the editor. As cutoff values for the model fit, we followed the criteria suggested by Schermelleh-Engel et al. (2003) with CFI ≥ 0.95 and RMSEA ≤ 0.08 for an acceptable fit. However, because the measurement models had rather few degrees of freedom and the sample size was small to medium in size, we decided to follow Kenny et al. (2015) being more liberal with regard to the RMSEA of the measurement models. From the measurement models and the structural model of the SSS-V, factor scores were estimated. Based on the factor scores, we specified two path models in which the dark traits and sensation seeking predicted (a) fire interest and (b) actual fire setting. Correlations between the predictor variables were allowed. Using path models instead of multiple regressions allowed the use of an MLR instead of an OLS estimator, potentially leading to more robust results. Furthermore, we calculated a logistic regression with fire setting as the outcome, as it was assessed dichotomously.

## Results

Means, standard deviations, and correlations between all scales can be found in Table 1. The zero-order correlations show that fire setting was positively related to fire interest, psychopathy, all sadism facets, and the sensation seeking facet boredom susceptibility. Fire interest was also related to these traits and also to Machiavellianism and the other sensation seeking facets.

**Table 1:** Means, standard deviations, and correlations with confidence intervals of all study variables.

**Variable**	***M***	***SD***	**1**	**2**	**3**	**4**	**5**	**6**	**7**	**8**	**9**	**10**	**11**
1. FS	0.07	0.26											
2. FI	46.48	8.44	0.29^**^										
			[0.16, 0.40]										
3. Mach	2.91	0.55	0.13	0.19^**^									
			[−0.01, 0.26]	[0.06, 0.32]									
4. Narc	3.06	0.55	−0.03	0.04	0.23^**^								
			[−0.16, 0.10]	[−0.09, 0.17]	[0.10, 0.35]								
5. Psych	2.16	0.62	0.39^**^	0.49^**^	0.48^**^	0.39^**^							
			[0.27, 0.49]	[0.38, 0.58]	[0.37, 0.58]	[0.27, 0.49]							
6. VerbS	2.42	1.22	0.26^**^	0.42^**^	0.25^**^	0.18^**^	0.54^**^						
			[0.13, 0.38]	[0.31, 0.53]	[0.13, 0.37]	[0.05, 0.30]	[0.44, 0.63]						
7. PhysS	1.55	0.92	0.34^**^	0.56^**^	0.18^**^	0.15^*^	0.59^**^	0.58^**^					
			[0.21, 0.45]	[0.46, 0.65]	[0.05, 0.31]	[0.01, 0.27]	[0.50, 0.67]	[0.48, 0.66]					
8. VicaS	2.36	1.05	0.34^**^	0.68^**^	0.29^**^	0.09	0.54^**^	0.54^**^	0.60^**^				
			[0.21, 0.45]	[0.60, 0.74]	[0.16, 0.40]	[−0.04, 0.22]	[0.44, 0.63]	[0.44, 0.63]	[0.51, 0.68]				
9. BS	3.31	2.03	0.16^*^	0.24^**^	0.23^**^	0.24^**^	0.41^**^	0.41^**^	0.35^**^	0.31^**^			
			[0.03, 0.29]	[0.11, 0.36]	[0.10, 0.35]	[0.11, 0.36]	[0.29, 0.51]	[0.30, 0.51]	[0.23, 0.46]	[0.18, 0.42]			
10. DIS	4.76	2.42	0.10	0.25^**^	0.05	0.16^*^	0.27^**^	0.27^**^	0.16^*^	0.17^*^	0.27^**^		
			[−0.03, 0.23]	[0.13, 0.37]	[−0.08, 0.18]	[0.03, 0.29]	[0.15, 0.39]	[0.14, 0.39]	[0.03, 0.28]	[0.04, 0.29]	[0.15, 0.39]		
11. ES	6.09	1.84	0.04	0.21^**^	−0.17^*^	0.02	0.06	0.03	−0.04	0.05	0.06	0.36^**^	
			[−0.09, 0.17]	[0.08, 0.33]	[−0.29, −0.04]	[−0.11, 0.15]	[−0.08, 0.19]	[−0.11, 0.16]	[−0.17, 0.09]	[−0.08, 0.18]	[−0.07, 0.19]	[0.24, 0.47]	
12. TAS	6.10	2.77	0.07	0.18^**^	−0.03	0.16^*^	0.09	0.10	0.04	0.13^*^	0.14^*^	0.31^**^	0.38^**^
			[−0.06, 0.20]	[0.05, 0.30]	[−0.16, 0.10]	[0.03, 0.28]	[−0.04, 0.22]	[−0.04, 0.22]	[−0.09, 0.17]	[0.00, 0.26]	[0.01, 0.27]	[0.19, 0.43]	[0.26, 0.49]

*FS = Fire setting; FI = Fire interest; Mach = Machiavellianism; Narc = Narcissism; Psych = Psychopathy; VerbS = Direct verbal sadism; PhysS = direct physical sadism; VicaS = Vicarious sadism; BS = Boredom susceptibility; DIS = Disinhibition; ES = Experience seeking; TAS = Thrill and adventure seeking*.

*Values in square brackets indicate the 95% confidence interval for each correlation.*

* *p < 0.05,*

** *p < 0.01*.

Model fits of the models were acceptable (see Supplementary Table 1) with all except the models for fire interest and the three Dark Triad traits. To improve the fit of those models, we used modification indices and when we considered these to be also theoretically justified, we allowed for correlations (see Supplementary Table 2 for details). Results from the path models are shown in Table 2. Psychopathy and direct physical sadism were positively associated with fire setting and fire interest, while narcissism was negatively associated with fire interest (suppression). Vicarious sadism was positively associated with fire interest but not fire setting. All sensation seeking facets were not substantially associated with fire setting and fire interest beyond the Dark Tetrad. Overall, 52% of variance in fire interest and 19% in fire setting could be explained. The logistic regression (Table 3) showed a similar result to the path model with psychopathy being positively and narcissism being negatively associated with fire setting. The odds ratios indicate that the chance of fire setting increases by 5.22 for every increase of 1 unit on the psychopathy scale and for each unit increase in narcissism, the odds of setting a fire decreased from 1 to 0.42, when all other predictors were kept constant. In the logistic regression, 38% (Nagelkerkes *R*^2^) of variance could be explained.

**Table 2:** Standardized estimates from path models.

	**Fire setting**	***p*** **-value**	**Fire interest**	***p*** **-value**
**Dark Triad**				
Machiavellianism	−0.06	0.550	−0.02	0.736
Narcissism	−0.13	0.055	−0.16	0.003
Psychopathy	0.29	0.036	0.15	0.027
**Sadism**				
Direct verbal	−0.01	0.886	−0.04	0.476
Direct physical	0.20	0.007	0.28	<0.001
Vicarious	0.03	0.779	0.48	<0.001
**Sensation seeking**				
TAS	0.03	0.662	0.03	0.526
BS	−0.04	0.592	0.01	0.892
DIS	−0.06	0.387	−0.04	0.410
ES	−0.11	0.211	0.06	0.247

*TAS = Thrill and adventure seeking; BS = Boredom susceptibility; DIS = Disinhibition; ES = Experience seeking*.

*Significant estimates are highlighted in bold font. R^2^ for fire setting = 0.19, for fire interest = 0.52*.

**Table 3:** Logistic regression with fire setting as criterion variables.

	***B***	***SE***	***z*** **-value**	***p*** **-value**	**Odds ratio**
**Dark Triad**					
Machiavellianism	0.19	0.40	0.47	0.641	1.20
Narcissism	−0.87	**0**.**44**	−1.99	**0**.**046**	0.42
Psychopathy	1.65	**0**.**63**	2.60	**0**.**009**	5.22
**Sadism**					
Direct Verbal	0.00	0.48	−0.01	0.994	1.00
Direct Physical	0.75	0.55	1.35	0.176	2.11
Vicarious	−0.01	0.48	−0.02	0.987	0.99
**Sensation seeking**					
TAS	0.33	0.43	0.75	0.454	1.39
BS	−0.22	0.57	−0.39	0.697	0.80
DIS	−0.28	0.50	−0.56	0.575	0.75
ES	−0.35	0.50	−0.70	0.484	0.70

*TAS = Thrill and adventure seeking; BS = Boredom susceptibility; DIS = Disinhibition; ES = Experience seeking*.

*Significant estimates are highlighted in bold font. Nagelkerke's R^2^ = 0.38*.

## Discussion

In this study, we observed the relationship of the Dark Tetrad and sensation seeking with fire interest and fire setting in a non-clinical population. We found psychopathy and direct physical sadism to be significantly correlated with fire interest and fire setting, and also related to both outcomes in the path models which included all variables. Direct verbal sadism was positively correlated with fire interest and fire setting but unrelated to both outcomes in the path model. Similarly, the sensation seeking facets were positively correlated with fire interest but were not associated with fire interest in the path model. We will discuss these findings in the following.

### Fire Interest vs. Fire Setting

Fire interest and fire setting were positively correlated by *r* = .29. This moderate correlation is in line with the M-TTAF (Gannon et al., 2012), which suggests that fire interest is a factor important for some but not all fire setters. For example, someone following the grievance trajectory proposed by the model would more likely be motivated to set a fire by revenge or retribution than by fire interest. For someone else following the fire interest trajectory, fire interest or fire fascination may be paired with stress or thrill seeking and then be the crucial motivator to set a fire. However, the rather low base rate of fire setters in our sample could also explain the relatively small size of the correlation.

We found higher correlations of physical (*z* = 2.92, *p* < .001) and vicarious sadism (*z* = 5.89, *p* < .001) with fire interest compared to fire setting. For vicarious sadism, where the difference showed itself most clearly, this could be explained by the fact that watching a fire may already satisfy sadistic needs (see Grant and Kim, 2007; Tyler and Gannon, 2017), while the act of setting the fire is of less importance. For physical sadism, this may just be the result of the interrelation with vicarious sadism (*r* = .60), i.e., shared variance that is also shared with fire interest. This is also underlined by the results of the path models, where vicarious sadism was related to fire interest but not to fire setting while all other substantial relations were similar. Similarly, the sensation seeking facets disinhibition and experience seeking were significantly correlated with fire interest but not with fire setting. Again, watching a fire or playing with fire (i.e., fire interest) may already satisfy the drive for excitement and disinhibition without the need for setting a fire. Also, the scale used (higher end: *lovely, very exciting, or very nice*) in the fire interest measure may imply a somewhat inflated relation to sensation seeking.

### The Dark Tetrad

Our results for *psychopathy* provide further evidence for the close relation of impulsivity and fire setting (e.g., Wyatt et al., 2019). Psychopathy showed the strongest relation of the three Dark Triad traits to fire interest and fire setting and was also positively related to both outcomes in the path models and the logistic regression where all other variables were controlled for. With impulsivity as a core facet of psychopathy, this finding turned out as expected and provides evidence for the psychopathy fire setting link beyond previous clinical research (e.g., Thomson et al., 2015).

While *Machiavellianism* was positively correlated with fire interest, it was not relevant in the path model and unrelated to fire setting. We argued that Machiavellianism could be relevant to fire setting through the antisocial trajectory of the M-TTAF (Gannon et al., 2012) and similar antisocial behaviors shown in fire setters and individuals scoring high in Machiavellianism (Blanco et al., 2010) but this was not supported by the data.

However, this finding provides evidence in favor of distinguishing psychopathy and Machiavellianism (cf. Vize et al., 2018) due to differences in the nomological networks with fire setting being a further aspect which tends to share more variance with psychopathy than Machiavellianism (which is in line with findings on vandalism; Pfattheicher et al., 2019). With regards to fire interest, the difference between psychopathy and Machiavellianism was also found in the path model but only to some extend in the correlations, as the relationship of psychopathy with fire interest was higher than the relationship of Machiavellianism and fire interest (*z* = 3.60, *p* < .001).

*Narcissism* did not substantially correlate with fire interest and fire setting but showed a significantly negative association to fire interest in the path model and to fire setting in the logistic regression. Thus, a suppression occurred, and we will not interpret the according estimates. This is in line with findings by Sleep et al. (2017), who found that narcissism lost its relations to several behavioral outcomes when the Dark Triad was entered in a regression and scores were residualized. They argued that narcissism loses its connection to antagonism, which is a core aspect of the construct (Sleep et al., 2017) and the partial regression weight of narcissism should not be interpreted. Therefore, we focus on the correlations, which show no relation of narcissism to fire interest and fire setting.

The findings for *sadism* are in line with previous research by Pfattheicher et al. (2019) who showed that object related violence in form of vandalism for pleasure was related to all three sadism facets. In a multiple regression with the Dark Triad and the three sadism facets, only direct physical sadism (and psychopathy) was a significant predictor of vandalism which we also found in the path model for fire setting but not in the logistic regression. Pfattheicher et al. (2019) argued that individuals scoring high in sadism are in a state of dominance and control when they harm other individuals and might harm objects to achieve a sense control and domination as well. Thus, vandalism, fire interest, and fire setting share direct physical sadism as an important association, while vicarious sadism seems to be most relevant to fire interest.

### Sensation Seeking

All four sensation seeking facets were correlated with fire interest but only boredom susceptibility was correlated with fire setting. In the path model where it was controlled for all other variables, these relations disappeared. While the relationship of fire setting and *boredom susceptibility* was likely due to similar previous findings (e.g., Doley et al., 2011) and based on the M-TTAF (Gannon et al., 2012), we also assumed a relationship to *thrill and adventure seeking*. In the M-TTAF, thrill-seeking is understood as a potential motivator in the fire interest trajectory which was represented in the data by the relation of thrill and adventure seeking to fire interest but not to fire setting. Dickens et al. (2009) reported that excitement, which is mainly reflected in the *disinhibition* facet, as a motive for fire setting is rare but 9% (*n* = 81) in fire setters who set multiple fires reported feeling of tension and excitement compared to 1% (*n* = 86) of fire setters who did not set multiple fires, indicating that an excitement motive tends to be more common among recidivistic fire setters. This indicates that a link between disinhibition and fire setting is potentially small and may more likely be found in forensic samples. We were not aware of research looking at a potential relationship of *experience seeking* and fire interest or fire setting. The relationship of fire interest and experience seeking, which we found, might be explainable by the general curiosity for new and uncommon things which is inherent in most experience seeking items.

The relation of the sensation seeking facets with fire interest vanished in the multivariate analyses when we controlled for the Dark Tetrad. This shows that the overlap of sensation seeking with the Dark Tetrad (see also Vize et al., 2018) seems to include what both share with fire interest. At the content level, this might be explainable through risk propensity, which is positively related to the Dark Triad (Wehner et al., 2021), inherent in sensation seeking, and necessary when it comes to fire setting. Moreover, these results could inform prevention and clinical efforts. Our findings show that potential interventions should focus on the Dark Tetrad, not on sensation seeking, which does not appear to explain substantial variance beyond the Dark Tetrad, particularly beyond psychopathy and sadism.

### Limitations and Future Directions

Our study is one of the few studies, which have observed fire interest and fire setting in the general population and not in a clinical or forensic sample (see Blanco et al., 2010; Gannon and Barrowcliffe, 2012 as further exceptions). However, our sample only includes students, and the generalizability of our result may be limited. We added several correlations to the measurement models of the three Dark Triad traits and fire interest to improve the model fit, which needs replication in future studies. Furthermore, fire setting was assessed with only one item, which did not allow for a distinction between multiple and one-time fire setters, and provided no information about the severity of the fires set. Also, our sample was only small to medium in size, but we could assume to find stable correlations of ρ = .10 with a confidence level of 95% and a width of the corridor of stability of *w* < .15 (Schönbrodt and Perugini, 2013) for fire interest. Finally, we used faceted measures to assess sadism and sensation seeking but not for the Dark Triad traits. Future research could utilize the Dark Triad facet level to get a more comprehensive picture.

## Conclusion

Knowledge on the origins of fire interest and fire setting is relatively little, however, multiple factors such as childhood experiences or human evolution that lead to cognitive patterns surrounding fire (Fessler, 2006) may play a role. Fire setting and fire interest have been hypothesized to be influenced by personality traits such as impulsivity and thrill-seeking (Gannon et al., 2012). Our study indicates that, in a non-clinical sample, psychopathy and direct physical sadism could be a new addition to these factors improving the understanding of fire interest and fire setting. For fire interest, vicarious sadism also played a crucial role and appeared to be the best predictor in distinguishing between a mere fire interest and actual fire setting. Furthermore, this study suggests, following the assumption of Pfattheicher et al. (2019), that sadism is not only representative of interpersonal aggression, but is also able to predict object-related aggression. The other constructs of the Dark Tetrad and sensation seeking do not seem to play an important role in the prediction of fire setting and fire interest beyond psychopathy and sadism. Finally, these results further strengthen the notion of separability between psychopathy and Machiavellianism.

## Data Availability Statement

The datasets presented in this study can be found in online repositories. The names of the repository/repositories and accession number(s) can be found at: https://osf.io/z9rgd/.

## Ethics Statement

Ethical review and approval was not required for the study on human participants in accordance with the local legislation and institutional requirements. The patients/participants provided their written informed consent to participate in this study.

## Author Contributions

LL came up with the idea for the study. SK collected the data. MZ and CW conducted the analyses. CW wrote the first version of the manuscript. All authors provided comments and suggestions and approved the final version of the manuscript.

## Funding

We acknowledge support by the German Research Foundation (DFG) and the Open Access Publication Fund of Humboldt-Universität zu Berlin.

## Conflict of Interest

The authors declare that the research was conducted in the absence of any commercial or financial relationships that could be construed as a potential conflict of interest.

## Publisher's Note

All claims expressed in this article are solely those of the authors and do not necessarily represent those of their affiliated organizations, or those of the publisher, the editors and the reviewers. Any product that may be evaluated in this article, or claim that may be made by its manufacturer, is not guaranteed or endorsed by the publisher.

## Footnotes

^1^Narcissism as discussed in this study covers only the grandiose aspect of narcissism (cf. Krizan and Herlache, 2018; Crowe et al., 2019).

^2^Analyses were not pre-registered.
