# Supplementary material for: Bringing Light Into the Dark: Associations of Fire Interest and Fire Setting With the Dark Tetrad
Source: Front Psychol. 2022 Jun 27;13:876575. doi: 10.3389/fpsyg.2022.876575 (PMC9272986; doi:10.3389/fpsyg.2022.876575)
Supplement: Supplementary file 1 [file Data_Sheet_1.doc]

**Supplement**

| Table S1  *Model Fits of Original Models* | | | | | | | |  |
| --- | --- | --- | --- | --- | --- | --- | --- | --- |
|  | CFI | RMSEA | RMSEA 90% CI | SRMR | χ^2^ | p | df | |
| Fire Interest | .765 | .177 | [.164; .191] | .125 | 612.271 | <.001 | 77 | |
| *Dark Triad* |  |  |  |  |  |  |  | |
| Machiavellianism | .881 | .107 | [.087; .128] | .076 | 123.412 | <.001 | 35 | |
| Narcissism | .929 | .074 | [.048; .099] | .058 | 59.397 | <.001 | 27 | |
| Psychopathy | .925 | .097 | [.073; .121] | .069 | 82.678 | <.001 | 27 | |
| *Sadism* |  |  |  |  |  |  |  | |
| Direct Verbal | .974 | .141 | [.104;.181] | .045 | 48.624 | <.001 | 9 | |
| Direct Physical | .991 | .115 | [.065; .171] | .041 | 19.726 | <.001 | 5 | |
| Vicarious | .986 | .083 | [.049; .118] | .042 | 35.323 | .001 | 14 | |
| *Sensation Seeking* |  |  |  |  |  |  |  | |
| Bifactor Model | .957 | .030 | [.021; .037] | .114 | 836.241 | <.001 | 700 | |
| TAS | .944 | .070 | [.047; .093] | .081 | 72.971 | <.001 | 35 | |
| BS | .866 | .051 | [.021; .075] | .099 | 54.775 | .018 | 35 | |
| DIS | .999 | .008 | [.000; .049] | .066 | 35.495 | .445 | 35 | |
| ES | .950 | .023 | [.000; .055] | .088 | 38.970 | .296 | 35 | |
| *Note*. TAS = thrill and adventure seeking; BS = boredom susceptibility; DIS = disinhibition; ES = experience seeking. WLSMV estimators were used. | | | | | | | |  |

| Table S2  *Model Fits of Modified Models* | | | | | | | |  |
| --- | --- | --- | --- | --- | --- | --- | --- | --- |
|  | CFI | RMSEA | RMSEA 90% CI | SRMR | χ^2^ | p | df | |
| Fire Interest^*^ | .916 | .110 | [.096; .124] | .090 | 309.613 | <.001 | 74 | |
| *Dark Triad* |  |  |  |  |  |  |  | |
| Machiavellianism^*^ | .949 | .082 | [.056; .108] | .056 | 61.973 | <.001 | 25 | |
| Narcissism^*^ | .946 | .065 | [.038; .092] | .053 | 50.609 | <.001 | 26 | |
| Psychopathy^*^ | .959 | .073 | [.046; .099] | .055 | 39.231 | <.001 | 26 | |
| *Sadism* |  |  |  |  |  |  |  | |
| Direct Verbal | .974 | .141 | [.104;.181] | .045 | 48.624 | <.001 | 9 | |
| Direct Physical | .991 | .115 | [.065; .171] | .041 | 19.726 | <.001 | 5 | |
| Vicarious | .986 | .083 | [.049; .118] | .042 | 35.323 | .001 | 14 | |
| *Sensation Seeking* |  |  |  |  |  |  |  | |
| Bifactor Model | .957 | .030 | [.021; .037] | .114 | 836.241 | <.001 | 700 | |
| *Note.* TAS = thrill and adventure seeking; BS = boredom susceptibility; DIS = disinhibition; ES = experience seeking. WLSMV estimators were used.  ^*^ To improve model fit we allowed the following correlations: *Machiavellianism*: item 1 (“It’s not wise to tell your secrets.”) and 7 (“There are things you should hide from other people to preserve your reputation.”), item 5 (“It’s wise to keep track of information that you can use against people later.”) and 6 (“You should wait for the right time to get back at people.”). *Narcissism*: item 5 („I like to get acquainted with important people.“) and 9 (“I insist on getting the respect I deserve”). *Psychopathy*: item 2 (“I avoid dangerous situations.”) and 7 (“I have never gotten into trouble with the law.“). *Boredom susceptibility*: item 5 (“I get bored seeing the same old faces.”) and 24 (“I prefer friends who are excitingly unpredictable”). *Fire interest*: item 1 (“Watching ordinary coal fire in an ordinary house.”) and 10 (“Watching bonfire outdoors, like on a bonfire night.”), item 3 (“Seeing firemen get equipment ready”) and 6 (“Seeing firemen hosing a fire”), item 4 (“Striking match to light a cigarette”) and 11 (“Having matches in your pocket”), item 9 (“Watching a person with clothes on fire”) and 10, item 10 and 11. | | | | | | | |  |
